# Coagulation- and fibrinolysis-related genes for predicting survival and immunotherapy efficacy in colorectal cancer

**DOI:** 10.3389/fimmu.2022.1023908

**Published:** 2022-11-30

**Authors:** Yanling Ma, Bofang Wang, Puyi He, Wenbo Qi, Ling Xiang, Ewetse Paul Maswikiti, Hao Chen

**Affiliations:** ^1^ Second Clinical Medical College, Lanzhou University, Lanzhou, China; ^2^ Department of Cancer Center, Lanzhou University Second Hospital, Lanzhou, China

**Keywords:** colorectal cancer, coagulation and fibrinolysis system, immune infiltration, risk score model, prognosis

## Abstract

**Background:**

Colorectal cancer (CRC) is a common cancer and has a poor prognosis. The coagulation system and fibrinolysis system are closely related to the progression of malignant tumors and is also related to the immunotherapy of malignant tumors. Herein, we tried to predict survival and the immunotherapy effect for patients with CRC using a novel potential prognostic model.

**Methods:**

Through online data of TCGA and GEO, we screened significantly differentially expressed genes (DEGs) to construct a prognostic model, followed by obtaining immune-related genes (IRGs) from the ImmPort database and coagulation- and fibrinolysis-related genes (CFRGs) from the GeneCards database. The predictive power of the model is assessed by Kaplan–Meier survival curves as well as the time-dependent ROC curve. Moreover, univariate and multivariate analyses were conducted for OS using Cox regression models, and the nomogram prognostic model was built. In the end, we further studied the possibility that CXCL8 was associated with immunocyte infiltration or immunotherapy effect and identified it by immunohistochemistry and Western blot.

**Results:**

Five DEGs (CXCL8, MMP12, GDF15, SPP1, and NR3C2) were identified as being prognostic for CRC and were selected to establish the prognostic model. Expression of these genes was confirmed in CRC samples using RT-qPCR. Notably, those selected genes, both CFRGs and IRGs, can accurately predict the OS of CRC patients. Furthermore, CXCL8 is highly correlated with the tumor microenvironment and immunotherapy response in CRC.

**Conclusion:**

Overall, our established IRGPI can very accurately predict the OS of CRC patients. CXCL8 reflects the immune microenvironment and reveals the correlation with immune checkpoints among CRC patients.

## Introduction

Colorectal cancer (CRC) is of clinical interest because it is the third most common cancer in the world, and its incidence rate increased in developing countries such as China (1, 2). In addition, metastatic CRC patients remain with a poor prognosis with a median 5-year survival (1). A number of research indicated that the development of cancer is closely related to the immune system, especially the tumor microenvironment in the human body (3), and multiple mechanisms have evolved from cancer, such as escaping immune surveillance, resulting in the deficiency of antitumor immune responses (4). Besides standard conventional treatments, cancer immunotherapy is extensively studied and has become a promising treatment for the past few years, utilizing the immune system in combating cancer cells. Immunotherapy has various types, including immune checkpoint inhibitors (ICIs) and adoptive cell transfer. However, only a small proportion of patients benefit from immunotherapy due to its varying efficacy (5). Immune cells are the base of immunotherapy; thus, investigating immune cell infiltration in CRC is necessary. With the recent technological advances in RNA sequencing (RNA-seq) and mass cytometry, it is practicable to deeply understand the functional diversities of tumor-infiltrating immune cells.

The development of CRC involves three main types of genomic instability, namely, MSI, CIMP, and CIN (6). Mutation of several genes becoming cancer suppressors, such as TP53, SMAD2/4, and APC, can be observed in CIN colorectal tumors (7). Furthermore, MLH1 promoter hypermethylation is associated with CIMP and MSI tumors (8). For the past few years, CRC molecular subtyping systems can be divided into four CMS, proposed to explore the genetic and epigenetic phenomena (9, 10). CMS1 was regularly characterized by possessing BRAF mutations and displayed a high tumor mutation (11). Moreover, CXCL9 and CXCL10 are strongly involved in T-cell chemotaxis in CMS1 tumors; the gene coding CXCL13 is involved in the constitution of tertiary lymphoid structures (12). Nevertheless, these CMS1 tumors may overcome immune escape achieved by checkpoint overexpression, such as CTLA-4, LAG3 (CD223), PD-1, or PD-L1. Coagulation and fibrinolysis-related genes (CFRGs) are involved in several progressions of cancer and are directly correlated with tumor growth, invasion, and migration. Notably, they are recognized as diagnostic and therapeutic targets (13–17). Coagulation and immune systems are composed of an interconnected network that performs an effective response to anti-damage and anti-pathogen invasion (18). CXCL8 comprises both chemokines and CFRGs. CXCL8 plays a key role in the inflammation process, and certain studies suggested that inflammation is part of innate immunity (19); moreover, CXCL8 promotes immune evasion by inducing the expression of PD-L1 in gastric cancer, as previous studies reported (20). The secretion of CXCL8 and CXCL1 drives colorectal cancer cell migration (21).

Although CRC characteristics have been described based on CFRGs and IRGs, there is an urgent need for more comprehensive indicators to predict the survival rate of CRC patients and the efficacy of immunotherapy. Therefore, we constructed an IRGPI from CFRGs and IRGs based on the online database and bioinformatics. Furthermore, we assessed the effect on the prognostic or predictive value of IRGPI and identified the role of CXCL8 in immune infiltration in CRC.

## Materials and methods

### Data download and collection of sample information

Firstly, to identify the transcriptomic data of CRC samples, we extensively searched the datasets from the GEO database and downloaded three datasets through the geoquery package (22) in the R software. The GSE110223 dataset included 13 pairs of patients with confirmed colorectal adenocarcinomas and normal colon tissues. The GSE110224 and GSE113513 datasets contained 17 and 14 cases of CRC and matched normal samples, respectively. We also obtained the clinical information and mRNA data (including 480 colon cancer tissues and 41 normal tissues) from the TCGA data portal. The total TCGA queue was equally classified into two groups, namely, TCGA validation set 1 (*n* = 219) and TCGA validation set 2 (*n* = 219). Furthermore, 496 CFRGs and 2,484 IRGs were acquired respectively from the GeneCards and Analysis Portal (ImmPort) database (23).

### Screening of differentially expressed coagulation and fibrinolysis-related genes and immune−related genes

Before screening the differentially expressed genes (DEGs), all datasets were first normalized using the “limma” package and PCA diagrams were drawn to evaluate the differences between groups. We finally identified the DEGs with the cutoff conditions of |log_2_FC| >1 and *p* < 0.05. The volcano plot of DEIRGs and Venn diagram were plotted by the R “ggplot2” package for visualization.

### Functional enrichment analysis

To elucidate the latent functions of DECFRGs, we worked with the org.Hs.eg.db package to transfer gene ID and used the clusterProfiler package (24) (version: 3.14.3) to clarify the potential functional enrichment analysis. Enrichment analysis includes KEGG pathway analyses and GO analyses, including BP, CC, and MF. *p* < 0.05 denotes statistically significant differences. To visualize the above enrichment analysis, the ggplot2 package was used to represent the top items per ontology with bubble diagram and KEGG pathways with a network diagram.

### Establishment of the prognostic model

Univariate Cox regression analysis was performed to calculate the association between predicted gene expression levels and OS of CRC patients. Genes were suggested to provide a meaningful prognostic latent capacity at a *p*-value <0.05. Then, we built a prognostic model for OS based on the LASSO regression *via* the R package “glmnet” (25, 26). Finally, we established a survival risk model that divided the patients into low-risk and high-risk groups after delimiting prognostic genes and their LASSO coefficients. Kaplan–Meier plots were used to evaluate the prognosis of CRC patients with a cutoff value of *p* < 0.05. Additionally, the time-dependent ROC curve at specified time points (1 year, 3 years, and 5 years) was conducted to access the accuracy and sensitivity of survival prediction. The risk score = confident * expression of gene1 + confident * expression of gene2 + confident * expression of gene3 + confident * expression of gene4 +… + confident *expression of gene N.

### Immune cell infiltration

TIMER is a tool to analyze and visualize the correlation between levels of immune infiltrate and diverse tumor types. The correlation between CXCL8 expression and tumor-infiltrating immune cells was investigated through TIMER and ssGSEA in the GSVA package. Next, we used the R package “Estimate” to obtain the three scores of CXCL8, including immune score stromal score, stromal score, and estimate score. The TISIDB database provided us the associations for CXCL8 with immunomodulators, lymphocytes, and drugs. Furthermore, immune checkpoint molecules play a crucial role in cancer therapy. Thus, we explored the relationship between CXCL8 and the CTLA-4 inhibitor ipilimumab, PD-1 inhibitors, PD-L1 inhibitors, and so on.

### Collection of tumor tissues and RT-qPCR

Human colorectal tumor specimens were provided by the Department of Oncology Surgery, The Second Clinical Medical College of Lanzhou University. All experiments involving human tissues complied with the principles of the Declaration of Helsinki and have been approved by the Institutional Review Board of the Second Hospital of Lanzhou University. TRIzol was used to obtain total RNA extraction. SuperScript™ III First-Strand Synthesis SuperMix was used to synthesize for qRT-PCR. Then, we perform real-time PCR with Power SYBR^®^ Green PCR Master Mix kit. The primers used are shown in **Supplementary Table 2**.

### Western blotting

Extraction of total protein from CRC cells and tissue samples is carried out by RIPA and a protease inhibitor 99:1 mixture. After centrifugation, the collected supernatant was further analyzed. Protein concentration was determined by BCA assay, analysis, and denaturation. Then, total protein was loaded onto 12% PAGE gels and transferred onto PVDF membranes. Afterwards, the membrane was blocked with 5% nonfat milk for 2 h at 37°C on a shaker and incubated with the primary CXCL8/IL-8 antibodies and GAPDH antibodies at 4°C overnight on a shaker. Then, the secondary anti-mouse or anti-rabbit antibodies were used to incubate the membranes at room temperature for 1 h, followed by visual analysis using the ECL developer on the Tianneng exposure imaging system (TANO, Shanghai, China).

### Immunohistochemistry

Whole tissues were paraffin embedded and serially cut into 4-μm-thick sections, The sections were baked in an oven, dewaxed in xylene, soaked in alcohol, and washed with distilled water. After antigen retrieval, sample peroxidase activity was blocked with 3% H_2_O_2_. Afterwards, primary antibodies (anti-CXCL8/IL-8, CD3, CD4, CD8, CD20, CD56, CD68, and CD163) were used to incubate sections overnight at 4°C. The next day, sections were incubated with anti-rabbit antibody for 30 min. Last, sections were stained with 3,3’-diaminobenzidine.

### Statistical analysis

Statistical analyses were made with the GraphPad Prism 8.0.1 software. Statistical significance was determined using unpaired two-tailed Student’s *t*-test: *p < 0.05.

## Results

### Data preprocessing and DEG identification

**Figure 1:**
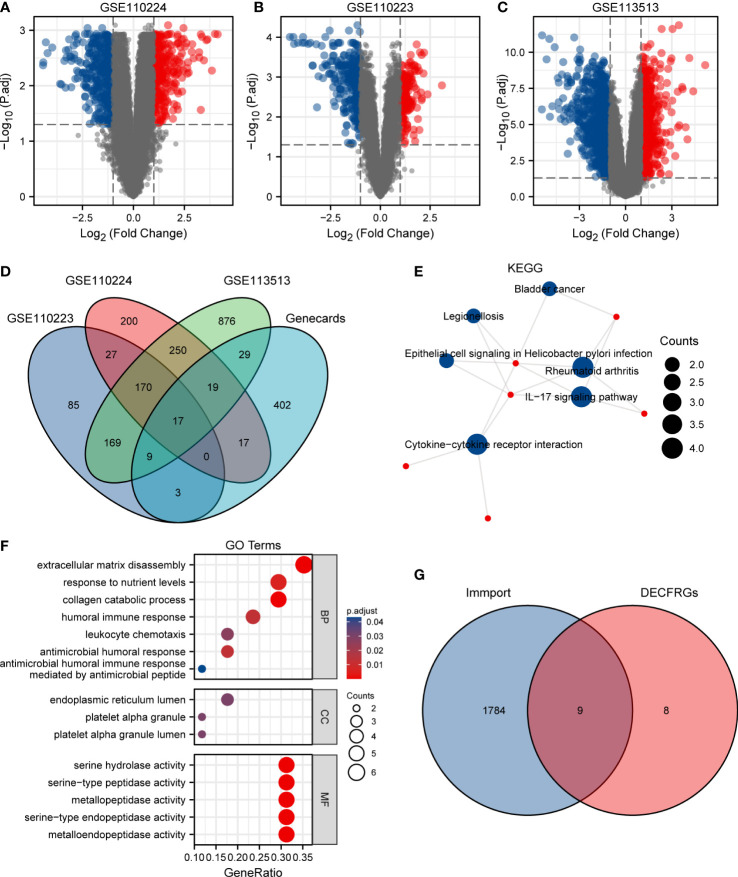
Identification of DEGs in CRC. **(A)** Volcano plot of DEGs between CRC and normal tissues in GSE110224. **(B)** Volcano plot of DEGs between CRC and normal tissues in GSE110223. **(C)** Volcano plot of DEGs between CRC and normal tissues in GSE113513. **(D)** Venn diagram visualizing the intersections of DEGs with CFRGs. **(E)** KEGG enrichment analysis of 17 DECFRGs. **(F)** GO enrichment analysis of 17 DECFRGs. **(G)** Venn diagram visualizing the intersections of DECFRGs with IRGs.

As shown in **Figure 1A**, 700 DEGs, namely 291 upregulated genes and 409 downregulated genes, were identified in the GSE110224 dataset. Meanwhile, we screened the DEGs in the GSE110223 dataset with a threshold at |logFC| > 1 and adjusted *p* at < 0.05, for a total of 480 genes, 182 of which were upregulated and 298 were downregulated (**Figure 1B**). In the GSE113513 dataset, there are 1,539 DEGs (namely 585 upregulated genes and 954 downregulated genes) (**Figure 1C**). The threshold was set at |logFC| > 1 and adjusted *p* at < 0.05. After intersecting the DEGs with CFRGs, we finally came to 17 genes (**Figure 1D**). To explore the biological function of these 17 DECFRGs, we performed GO and KEGG enrichment analyses using the clusterProfiler package in R.

KEGG enrichment analysis indicated that the signal pathways most related to the DECFRGs were rheumatoid arthritis, cytokine–cytokine receptor interaction, and IL-17 signaling pathway (**Figure 1E**). Furthermore, the top 4 enriched terms in the aspect of BP were extracellular matrix disassembly, response to nutrient levels, collagen catabolic process, and humoral immune response. The enriched GO items included endoplasmic reticulum lumen, platelet alpha granule lumen, and platelet alpha granule in the CC group. DECFRGs were mostly enriched in serine-type endopeptidase activity, metallopeptidase activity, and cytokine activity in MF (**Figure 1F**). Detailed GO and KEGG information is provided in **Supplementary Table 1**. Finally, nine DEIRGs were identified through the Venn diagram (**Figure 1G**).

### Construction of IRGPI for CRC and identification of DEGs

**Figure 2:**
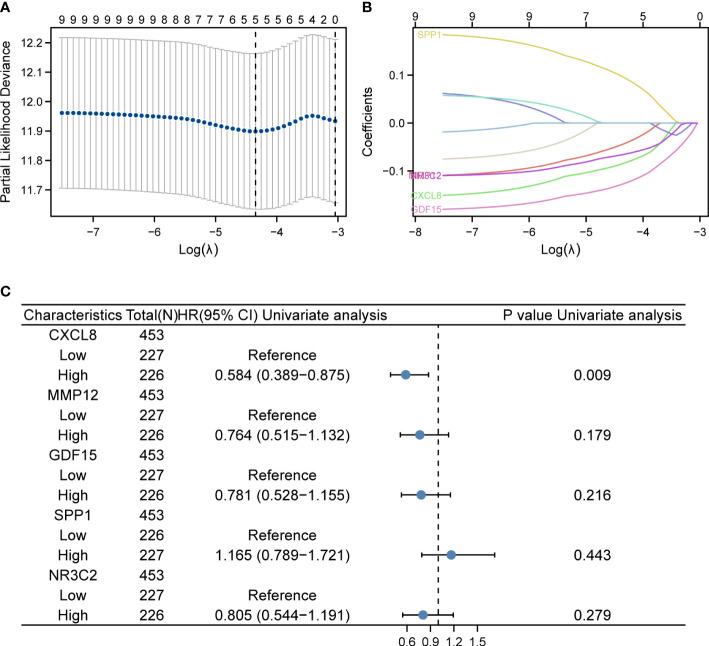
LASSO regression and risk score calculation. **(A)** Coefficient value of DEIRGs. **(B)** Partial likelihood deviance of DEIRGs. **(C)** Forest plot demonstrating the multivariable Cox model results of each gene in DEIRGs.

In the TCGA dataset containing 521 patients, duplicate samples and non-clinical samples were excluded. Univariate analysis was conducted and LASSO regression was adopted to conduct IRGPI. Lambda.min was selected to produce the model with a higher accuracy rate (**Figure 2A**); thus, we ended up with five prognostic genes: CXCL8, MMP12, GDF15, SPP1, and NR3C2 (**Figure 2B**). Univariate analysis showed that CXCL8 is remarkably related to OS of patients with COAD (**Figure 2C**). After multiplying gene expression levels with LASSO coefficients, we obtained the risk score = (−0.0843825) * expression of CXCL8 + (−0.043955) * expression of MMP12 + (−0.127046) * expression of GDF15 + 0.09601238 * expression of SPP1 + (−0.0656746) * expression of NR3C2.

**Figure 3:**
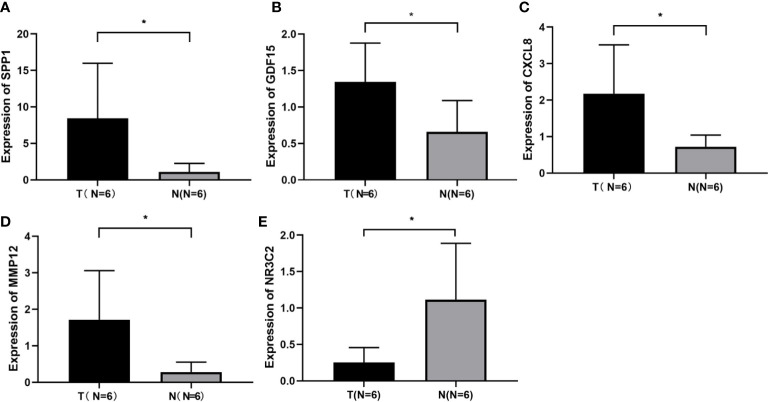
Identification of screened DEGs **(A)** The expression of gene of SSP1 in the indicated groups. **(B)** The expression of gene of GDF15 in the indicated groups. **(C)** The expression of gene of CXCL8 in the indicated groups. **(D)** The expression of gene of MMP12 in the indicated groups. **(E)** The expression of gene of NR3C2 in the indicated groups. *p< 0.05.

The expression levels of these selected four genes were significantly increased in tumor tissue than normal tissue, and another gene had a lower expression compared to normal samples (**Figure S1**). To confirm the expression of hub genes, we also collected tumor and normal tissues from Lanzhou Second Hospital and performed RT-qPCR. The results were consistent with online data. The results suggested that CXCL8, MMP12, GDF15, and SPP1 had a higher expression, while NR3C2 had a lower expression compared with the normal group (**Figure 3**).

### IRGPI predicts survival of CRC patients

**Figure 4:**
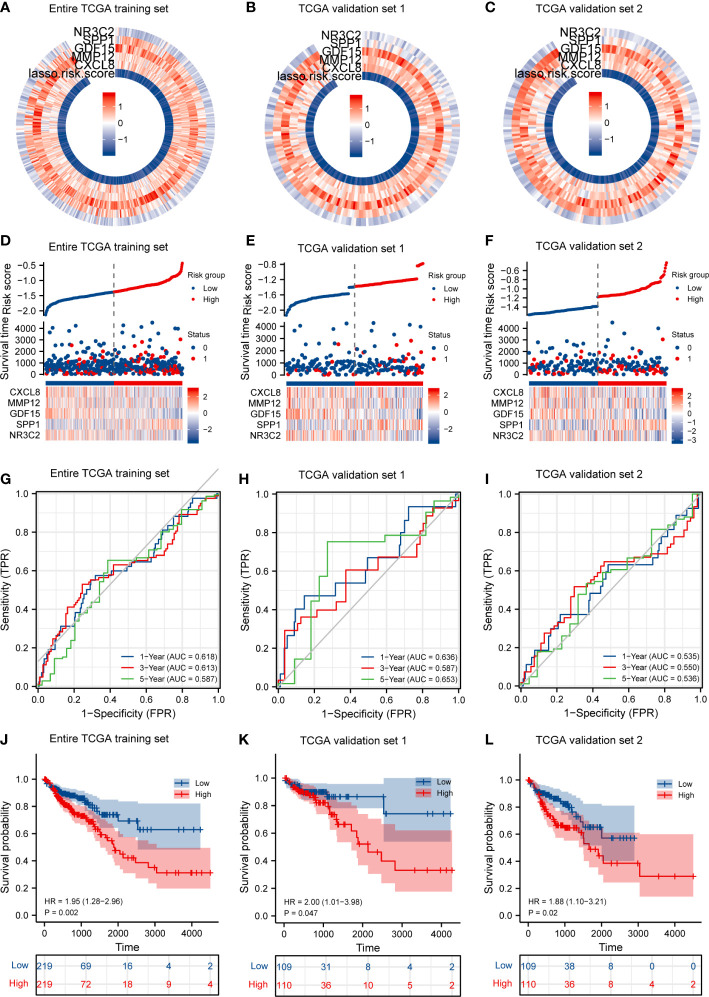
IRGPI accurately predicts survival of CRC patients and risk score validation. **(A)** Risk score and expression of five DEGs in the TCGA training set. **(B)** Risk score and expression of five DEGs in TCGA validation set 1. **(C)** Risk score and expression of five DEGs in TCGA validation set 2. **(D)** Risk score distribution, survival status, and expression of five IRGs for patients in low- and high-risk groups in the TCGA training dataset. **(E)** Risk score distribution, survival status, and expression of five IRGs for patients in low- and high-risk groups in TCGA validation set 1. **(F)** Risk score distribution, survival status, and expression of five IRGs for patients in low- and high-risk groups in TCGA validation set 1. **(G)** Time-dependent ROC curve analyses of risk score in the TCGA test set. **(H)** Time-dependent ROC curve analyses of risk score in TCGA validation set 1. **(I)** Time-dependent ROC curve analyses of risk score in TCGA validation set 2. **(J)** Risk score and survival probabilities in the TCGA test set. **(K)** Risk score and survival probabilities in TCGA validation set 1. **(L)** Risk score and survival probabilities in TCGA validation set 2.

First, according to the median value of IRGPI risk score, we calculated each patient’s risk score and classified the critical values of the high- and low-risk group in the TCGA training set (**Figures 4A, D**), in TCGA validation set 1 (**Figures 4B, E**), and in TCGA validation set 2 (**Figures 4C, F**), and the IRGPI thresholds are −1.354126396, −1.460113703, and −1.248139089, respectively. In the training set, patients in the high-risk group had a significantly poorer OS, and those included in the low-risk group had a higher OS (**Figure 4J**, *p* < 0.05). As shown in **Figure 4G**, the time-dependent ROC curve respectively indicated an AUC at 1 year, 3 years, and 5 years of 0.618, 0.613, and 0.587, respectively. When dividing the entire TGCA dataset into validation sets, we discovered that IRGPI also presented a high correctness of the predictive OS in TCGA validation set 1 and TCGA validation set 2. The validation datasets were also applied to the training set, and the AUC for 1-year, 3-year, and 5-year survival was 0.636, 0.587, and 0.653 in TCGA validation set 1, respectively (**Figures 4H, K**). Finally, validation set 2 had a *p*-value of <0.02 and an AUC of 0.535, 0.550, and 0.536 for 1-year, 3-year, and 5-year survival, correspondingly (**Figures 4I, L**). The outcome suggested that IRGPI could be a good dependable target and better than other features.

### Prognostic analysis for CRC

**Figure 5:**
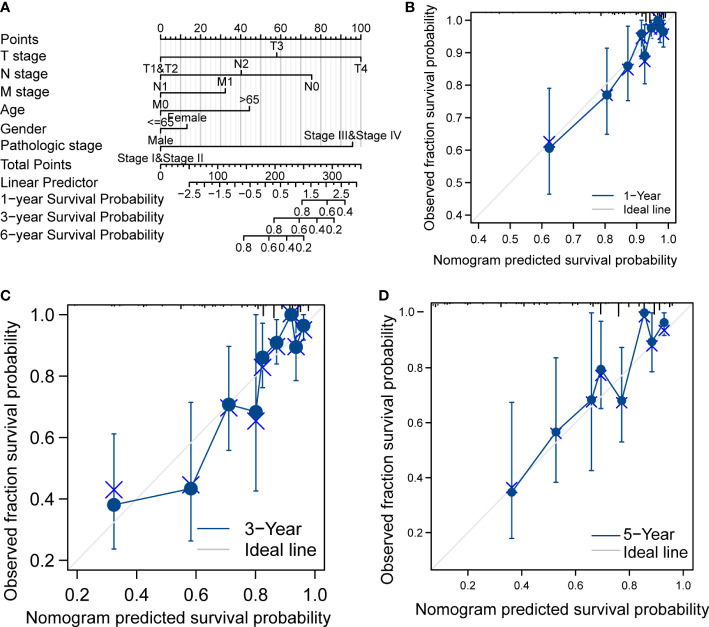
Prognostic analysis of CRC. **(A)** Nomogram of the prognostic model in the entire TCGA dataset. **(B)** Calibration of the 1-year prognostic model in the entire TCGA set. **(C)** Calibration of the 3-year prognostic model in the entire TCGA set. **(D)** Calibration of the 6-year survival in the entire TCGA set.

In **Table 1**, we explored the relationship between clinicopathologic factors and OS *via* univariate analyses. The results demonstrated that there was a significant relationship between clinicopathologic factors (including age, T/N/M stage, CEA levels, and OS; *p* < 0.05). Additionally, we built a prognostic nomogram to forecast the survival risk of each CRC patient (**Figure 5A**). Although there was a good consistency between predictive and actual 1-year survival in the entire TCGA set, there was a bad consistency between predictive and actual 3- and 5-year survival (**Figures 5B–D**).

**Table 1:** Univariate Cox regression analyses and other clinicopathologic factors for OS in the entire TCGA set.

Characteristics	Total (*N*)	Univariate analysis			Multivariate analysis		
		HR	95% CI	*p*-value	HR	95% CI	*p*-value
Age	477						
≤65	194						
>65	283	1.610	1.052–2.463	0.028	0.641	0.000–Inf	1
Gender	477						
Female	226						
Male	251	1.101	0.746–1.625	0.627			
T stage	476						
T1&T2	94						
T3&T4	382	3.072	1.423–6.631	0.004	0.000	0.000–Inf	0.999
N stage	477						
N0	283						
N1	108	1.681	1.019–2.771	0.042		0.000–Inf	0.999
N2	86	4.051	2.593–6.329	<0.001		0.000–Inf	1
M stage	414						
M0	348						
M1	66	4.193	2.683–6.554	<0.001	0.003	0.000–Inf	1
Pathologic stage	466						
Stage I	81						
Stage II	186	2.035	0.785–5.273	0.143		0.000–Inf	0.999
Stage III	133	3.683	1.436–9.448	0.007	0.006	0.000–Inf	1
Stage IV	66	9.294	3.608–23.936	<0.001	1.000	0.000–Inf	1
CEA level	302						
≤5	195						
>5	107	3.128	1.788–5.471	<0.001	0.000	0.000–Inf	1
Primary therapy outcome	250						
PD	25						
SD	4	0.930	0.120–7.183	0.944	1.000	1.000–1.000	
PR	13	0.271	0.062–1.191	0.084	1.000	1.000–1.000	
CR	208	0.087	0.044–0.173	<0.001	0.000	0.000–Inf	0.999
Residual tumor	373						
R0	345						
R1	4	1.410	0.330–6.014	0.643	1.000	1.000–1.000	
R2	24	6.412	3.378–12.172	<0.001	0.001	0.000–Inf	1
Perineural invasion	181						
No	135						
Yes	46	1.940	0.982–3.832	0.056	1335724.493	0.000–Inf	1
Lymphatic invasion	433						
No	265						
Yes	168	2.450	1.614–3.720	<0.001	0.840	0.000–Inf	1
History of colon polyps	407						
No	262						
Yes	145	0.741	0.442–1.242	0.255			
Colon polyps present	249						
No	162						
Yes	87	1.324	0.738–2.373	0.346			

### CXCL8 positively correlates with immune cell infiltration in CRC

**Figure 6:**
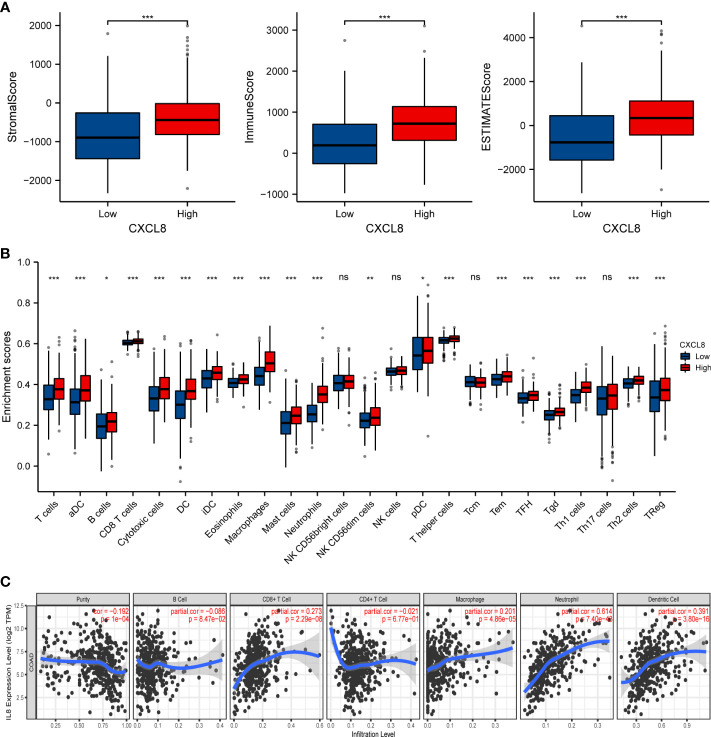
The relationship of immune cell infiltration with CXCL8 level in THCA. **(A)** The correlation of CXCL8 expression level with immune score, stromal score, as well as ESTIMATE score. **(B)** Relationships among infiltration levels of 24 immune cell types and CXCL8 expression profiles by ssGSEA. **(C)** Correlation of CXCL8 expression with immune infiltration level in CRC. *p < 0.05, **p < 0.01, ***p < 0.001.

CXCL8, as one of the genes in IRGPI, is one of the first and most intensively studied chemokines and is secreted by different cell types (27, 28). We have previously predicted that CXCL8 associated with immunity. Therefore, the correlation of CXCL8 with immune cell infiltration was evaluated. Firstly, the stromal score and immune score of CXCL8 showed a higher infiltration of stromal and immune cells when CXCL8 was highly expressed. Furthermore, the estimated score showed higher CXCL8 had a lower purity of tumor cells (**Figure 6A**, *p* < 0.0001). Then, we researched the relation between these immune cells and CXCL8. Among the 24 types of immune cells, the relative proportion of B cells, T cells, CD8 T cells, macrophages, and Treg had a significantly positive correlation with CXCL8 (**Figure 6B**). We further used TIMER to assess the immune infiltration levels of CXCL8. The outcomes suggested that there is a notable correlation between CXCL8 and tumor purity, CD8+ T cells, macrophages, neutrophils, and DCs (**Figure 6C**). Together, those results reveal the strong role of CXCL8 to reflect tumor microenvironment.

### Relationship between CXCL8 with immune molecules

**Figure 7:**
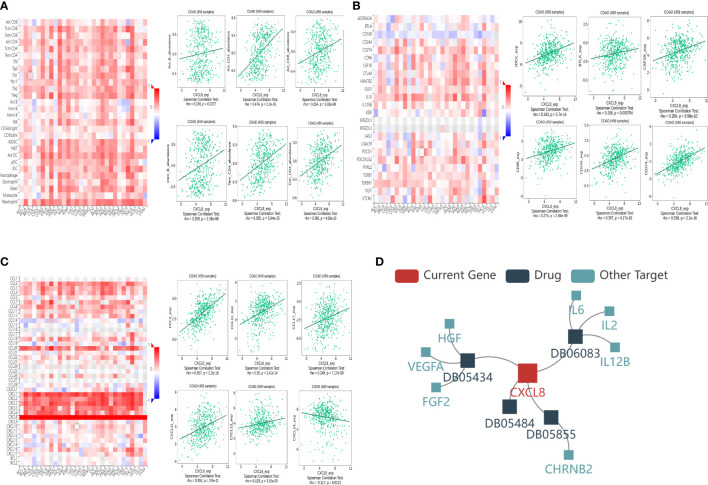
Associations of the CXCL8 expression level with lymphocytes, immunomodulators, chemokines, and drugs in COAD from the TISIDB database. **(A)** Correlations between abundance of tumor‐infiltrating lymphocytes (TILs) and CXCL8 (plus the six TILs with correlation). **(B)** Correlations between immunomodulators and CXCL8 (plus the six immunomodulators with correlation). **(C)** Correlations between chemokines and CXCL8 (plus the six chemokines with correlation, respectively). **(D)** Correlations between small-molecule drugs and CXCL8.

In order to further understand the correlation between CXCL8 and immune infiltration, we studied the relationship between CXCL8 expression and various immune characteristics, including TILs, immunomodulators, chemokines, and drugs. We obtained the correlation analysis between CXCL8 expression and several immune features from the TISIDB database. **Figure 7A** indicates the positive associations between CXCL8 and TILs, including Imm_B_abundance, Act_B_abundance, Act_CT4_abundance, Act_CT8_abundance, Tem_CD4_abundance, and Tcm_CD4_abundance. **Figure 7B** shows positive relationships between immune inhibitors including BTLA, IDO1, CSF1R, CD96, CD244, and CD274 with CXCL8. **Figure 7C** shows positive correlations between CXCL8 expression and chemokines, including CCL2_exp, CCL17_exp, CCL11_exp, CXCL11_exp, CXCL12_exp, and CCL14_exp. **Figure 7D** shows correlations between CXCL8 expression and drugs, including ABT510 targeting FGF2, HGF, VEGFA, CXCL8, Tapinaroftargeting IL2, IL6, IL12B, CXCL8, Rivanicline targeting CHRNB2,CXCL8 and MDX-018 targeting CXCL8, respectively. Thus, it could be confirmed that CXCL8 widely affected immune infiltration in the tumor microenvironment.

### Relation between CXCL8 and responses of immunotherapy

**Figure 8:**
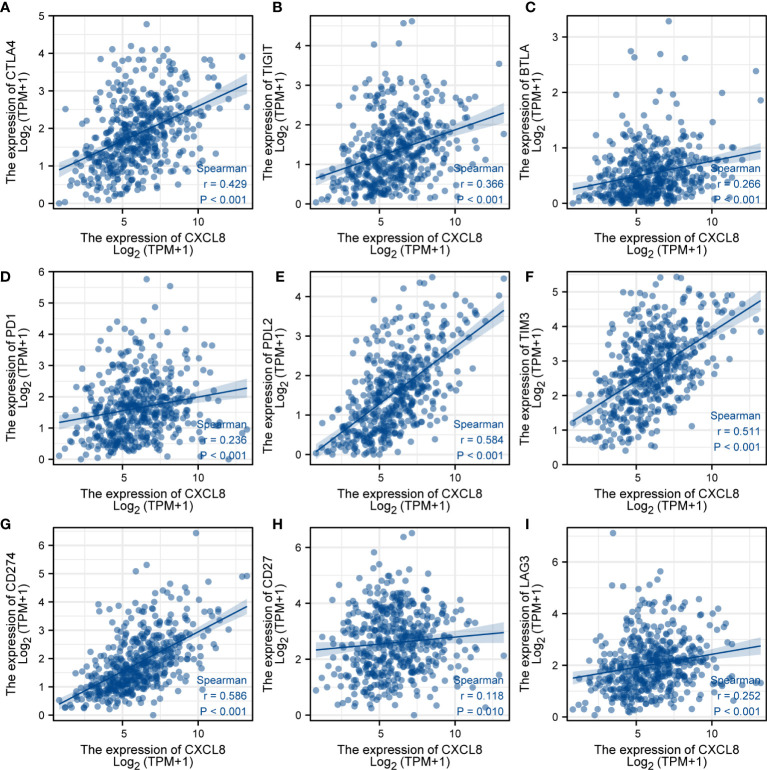
Analysis of immunotherapy responses. **(A)** Correlations of the expression of CXCL8 and CTLA-4. **(B)** Correlations of the expression of CXCL8 and TIGIT. **(C)** Correlations of the expression of CXCL8 and BTLA. **(D)** Correlations of the expression of CXCL8 and PD-1. **(E)** Correlations of the expression of CXCL8 and PD-L2. **(F)** Correlations of the expression of CXCL8 and TIM-3. **(G)** Correlations of the expression of CXCL8 and CD274. **(H)** Correlations of the expression of CXCL8 and CD27. **(I)** Correlations of the expression of CXCL8 and LAG3.

To further investigate the response of immune checkpoints, we conducted the relationship between CXCL8 and some immune checkpoints using the TCGA database. The major immune checkpoints include CTLA-4, PD-1, PD-L1, and PD-L2. After investigating the expression of some key immune checkpoints, the results indicated that the expression of CTLA-4 (**Figure 8A**), TIGIT (**Figure 8B**), BTLA (**Figure 8C**), PD-1 (**Figure 8D**), PD-L2 (**Figure 8E**), TIM-3 (**Figure 8F**), CD274 (**Figure 8G**), CD27 (**Figure 8H**) and LAG3 (**Figure 8I**) was positively related with expression of CXCL8 (p<0.05). Among these immune checkpoints, CXCL8 was highly correlated with PD-L2, TIM-3 and CD274. Taken together, these results suggested that CXCL8 had a strong response to immune response and immunotherapy.

### Validation of the role of CXCL8 in COAD

**Figure 9:**
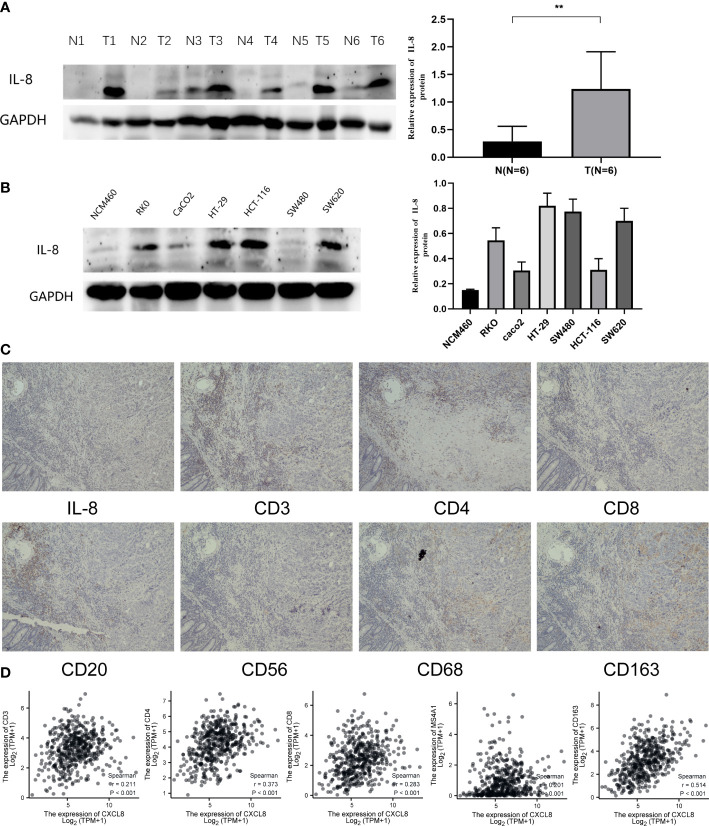
Expression of CXCL8 in COAD and the role of CXCL8 in immune infiltration. **(A)** WB revealed that CXCL8 expression was higher in tumor samples than normal samples. **(B)** Compared with NCM460 cells, CXCL8 had a higher expression in COAD cell lines, including RKO, CaCO_2_, HT-29, HCT-116, SW480, and SW620 cells. **(C)** IHC of CXCL8, CD3, CD4, CD8, CD20, CD56, CD68, and CD163 in the serial section of LIHC tissues (original magnification 100×). **(D)** CXCL8 expression was correlated with that of CD3, CD4, CD8, CD20 (MS4A1), and CD163 in COAD tissues (*p* < 0.05). **p < 0.01.

To identify the expression of CXCL8, we determined the CXCL8 protein expression level through Western blot both *in vivo* and *in vitro*. Consistent with the previous experiments, Western blot suggested that the protein expression of CXCL8 is higher in tumor tissues than normal tissues (**Figure 9A**, *p* < 0.05). Furthermore, we identified CXCL8 expression in several types of COAD cells. As shown in **Figure 9B**, CXCL8 levels were higher in all COAD cells than in control cells. To evaluate the role of CXCL8 in immune infiltration, we evaluated CXCL8 and various immune cell markers (including CD3, CD4, CD8, CD20, CD56, CD68, and CD163) *via* immunohistochemistry (IHC). As shown in **Figure 9C**. CXCL8 had a positive correlation with CD3, CD4, CD8, CD20 (MS4A1), and CD163 (**Figure 9D**, *p* < 0.05) in COAD; however, there is no significant correlation with CD56 and CD68. The results verified that CXCL8 was closely related with immune cell infiltration in COAD.

## Discussion

The evidence clearly indicated that tumor cells can activate the coagulation system, leading to the hypercoagulable state or prethrombotic state of malignant tumors. Thromboembolic disease is usually one of the earliest clinical symptoms of potential malignant tumors (29). It had been reported that the lungs, pancreas, and gastrointestinal tract exhibited a high incidence of thromboembolic disease (30, 31). Those patients with COAD accompanied by VTE have a high recurrence rate and significant mortality and morbidity (32, 33). The pathogenesis of the cancer-associated thrombosis is complex and multifactorial. Several risk factors, including clinical risk factors, fibrinolytic system, and the expression of procoagulant factors, activate the hemostatic system and contribute to thromboembolism (29, 34). The important relationship between colorectal cancer and thrombosis increases the possibility that the coagulation pathway becomes a therapeutic target.

In view of this, we obtained the 17 DECFRs in COAD and perform GO and KEGG enrichment analyses using bioinformatics tools. Our enrichment analysis indicated that those DECFRs were related to immune response. Thus, we subsequently screened nine genes both related to fibrinolytic system and immunity. Based on univariate and LASSO regression analyses, five out of nine genes were considered of prognostic value: CXCL8, MMP12, GDF15, SPP1, and NR3C2. We found that except for NR3C2, the expression of four of these genes increased. Based on the five genes, the risk score of each patient was calculated. When we categorized the samples based on the median of risk scores, the high-risk group manifested a clear survival disadvantage in the entire TCGA training set. The results were further validated in TCGA validation set 1 and TCGA validation set 2. In all datasets, the survival rate of high-risk patients was remarkably worse than that of low-risk patients.

In previous studies, CXCL8 (IL-8), as a major pro-inflammatory chemokine, mediates tumor immune escape through extracellular binding of two G-protein-coupled receptors (CXCR1 and CXCR2) (35). IL-8 is also an effective angiogenesis-promoting cytokine, which can promote the formation of new blood vessels and the growth and metastasis of tumors (31, 36). MMP12 consists of a number of matrix metalloproteinases (MMPs), which are a family of zinc-dependent endopeptidases. MMP12 was found to be correlated with a dismal prognosis in colon cancer patients’ serum (37). GDF15, released by many tissues, was identified as a member of the TGF-β superfamily (38). It was demonstrated that GDF15 was overexpressed in colorectal cancer (39). GDF15 promotes cell proliferation, migration, and invasion in colon cancer *via* the MAPK and PI3K signaling pathways (40). SPP1, a key extracellular matrix protein, is involved in colorectal cancer migration and invasion (41). Single-cell RNA sequencing (scRNA-seq) showed that *SPP1^+^* macrophages are enriched in tumor tissue, and the high expression of SPP1 contributed to the resistance to PD-L1 blocking immunotherapy (42). NR3C2, a nuclear transcription factor encoding the MR protein, *via* the AKT/ERK signaling pathway, inhibits the proliferation, migration, and invasion of colon cancer cells (43).

Cancer immunotherapy has become a promising strategy to treat cancer. Immune cells are the basis of cellular immunotherapy; thereby, to improve and develop effective therapeutic strategies for cancer treatment, it is vital to realize the immune infiltrates in the tumor microenvironment. Innate immune cells include NK cells, eosinophils, macrophages, and dendritic cells. The adaptive immunocytes contain B cells and T cells. They all have been proven to contribute to tumor progression and immunotherapy responses (5, 44). Immune cells will release cytokines to respond to cellular stresses such as tumorigenesis (45). IL-2, a typical cytokine therapy, can cause the patient’s cancer to subside (46). Recently, ICIs have entered medical practice and have become one of the principal immunotherapies nowadays (47). The most extensive used targets for ICIs are CTLA-4 and PD-L1. In our research, CXCL8 seems to be a valuable gene, because it had a significant relation to OS in univariate analysis. Furthermore, our supplementary figure shows that CXCL8 had a high diagnostic value in CRC. As previously reported, CXCL8 plays a key role in immunity of colon cancer by influencing the immune cells’ subsets; previous studies have shown that the role of tumor-infiltrating immune cells in human tumors is mainly focused on T cells, which are closely related to the current immunotherapy-related immune checkpoint mechanism (48). CXCL8 is significantly associated with infiltration of CD8+ T cells in colon cancer. As stated in the research, in an *in vivo* CRC mouse model, blocking the CXCL8–CXCR2 axis can decrease functional CD8+ T cell and DC infiltration into tumor sites, leading to the opposite effect of antitumor immunity, indicating that expression of CXCL8 is related to the infiltration of CD8+ T cells in colon cancer, and the process is completed by CXCL8–CXCR2 pathways and DC activation (28). Moreover, other studies show that expression of IL-8 can induce migration and recruitment of CXCR1 (+) CD8 (+) T cells in inflammatory areas (49) and using the Leptin trigger of the mouse model of colon cancer can promote the recruitment of T cells and enhance immune response. IL-8 plays an important role in this process (50); therefore, we think that there is a correlation between CXCL8 and the infiltration of CD8-positive T cells. Thus, we mainly researched the role of CXCL8 in immunotherapy. We found that the expression of CXCL8 was related to immune cells in COAD. Moreover, CXCL8 was highly positively correlated to ICIs, such as CTLA-4, PD-1, T CD27, and CD274. Thus, we forecasted that CXCL8 may be a major gene and provide insights into ICI therapy for COAD. IHC showed that CXCL8 is related to markers (CD3, CD4, CD8, CD20, and CD163) of immune cells. At present, the association between CXCL8 and tumor immunity and immunotherapy deserves much more attention.

In this study, the absence of sufficient clinical information on colorectal tumors prevents us from validating the prognostic model.

In summary, the risk score may predict the poor prognosis of patients with COAD. Nevertheless, CXCL8 plays a key role in immune therapy and immune cell infiltration, which provides the fundamental basis and a novel insight for further mechanism studies.

## Data availability statement

The original contributions presented in the study are included in the article/**Supplementary Material**. Further inquiries can be directed to the corresponding author.

## Ethics statement

This study was reviewed and approved by Committee of Medical Ethics Experts of the Second Hospital of Lanzhou University, approval number:2022A-369. Written informed consent was obtained from all participants for their participation in this study.

## Author contributions

YLM has made substantial contributions to the conception and design of the study, the acquisition of data, and the analysis and interpretation of data. HC edited the manuscript. BFW has been involved in drafting the manuscript and revising it. BW, PYH, WBQ, and LX provided technical assistance. EM provided writing assistance. All authors contributed to the article and approved the submitted version.

## Funding

This work was supported by the National Natural Science Foundation of China (No. 82160129), the Key Talents Project of Gansu Province (No. 2019RCXM020), the Key Project of Science and Technology in Gansu Province (19ZD2WA001), the COVID-19 Prevention and Control Technology Research Project of Lanzhou City (2020-XG-54), the Science and Technology Project of Chengguan District of Lanzhou City (2020SHFZ0039 and 2020JSCX0073), and the Cuiying Scientific and Technological Innovation Program of Lanzhou University Second Hospital (No. CY2017-ZD01). Outstanding Doctoral Program of Natural Science Foundation of Gansu Province (22JR5RA945), “Innovation Star” Project for Outstanding Graduate Students in Gansu Province (2022CXZX-162).

## Acknowledgments

We thank Dr. Ma Yu Ting for providing the database information and bioinformatic analysis.

## Conflict of interest

The authors declare that the research was conducted in the absence of any commercial or financial relationships that could be construed as a potential conflict of interest.

## Publisher’s note

All claims expressed in this article are solely those of the authors and do not necessarily represent those of their affiliated organizations, or those of the publisher, the editors and the reviewers. Any product that may be evaluated in this article, or claim that may be made by its manufacturer, is not guaranteed or endorsed by the publisher.

## Supplementary material

Supplementary Figure 1 The relationship of immune cell infiltration with CXCL8 level in THCA. **(A)** Overall survival analysis for CXCL8 in CRC patients was performed using the Kaplan–Meier plotter. **(B)** Survival-dependent receiver operating characteristic (ROC) curve validation of prognostic value of the prognostic index. **(C)** The correlation of CXCL8 expression level with immune score, stromal score, and ESTIMATE score. **(D)** Relationships among infiltration levels of 24 immune cell types and CXCL8 expression profiles by ssGSEA. **(E)** Correlation of CXCL8 expression with immune infiltration level in CRC. *p < 0.05, **p < 0.01, ***p < 0.001.
