# Supplementary figures and images for: Coagulation- and fibrinolysis-related genes for predicting survival and immunotherapy efficacy in colorectal cancer
Source: Front Immunol. 2022 Nov 30;13:1023908. doi: 10.3389/fimmu.2022.1023908 (PMC9748552; doi:10.3389/fimmu.2022.1023908)

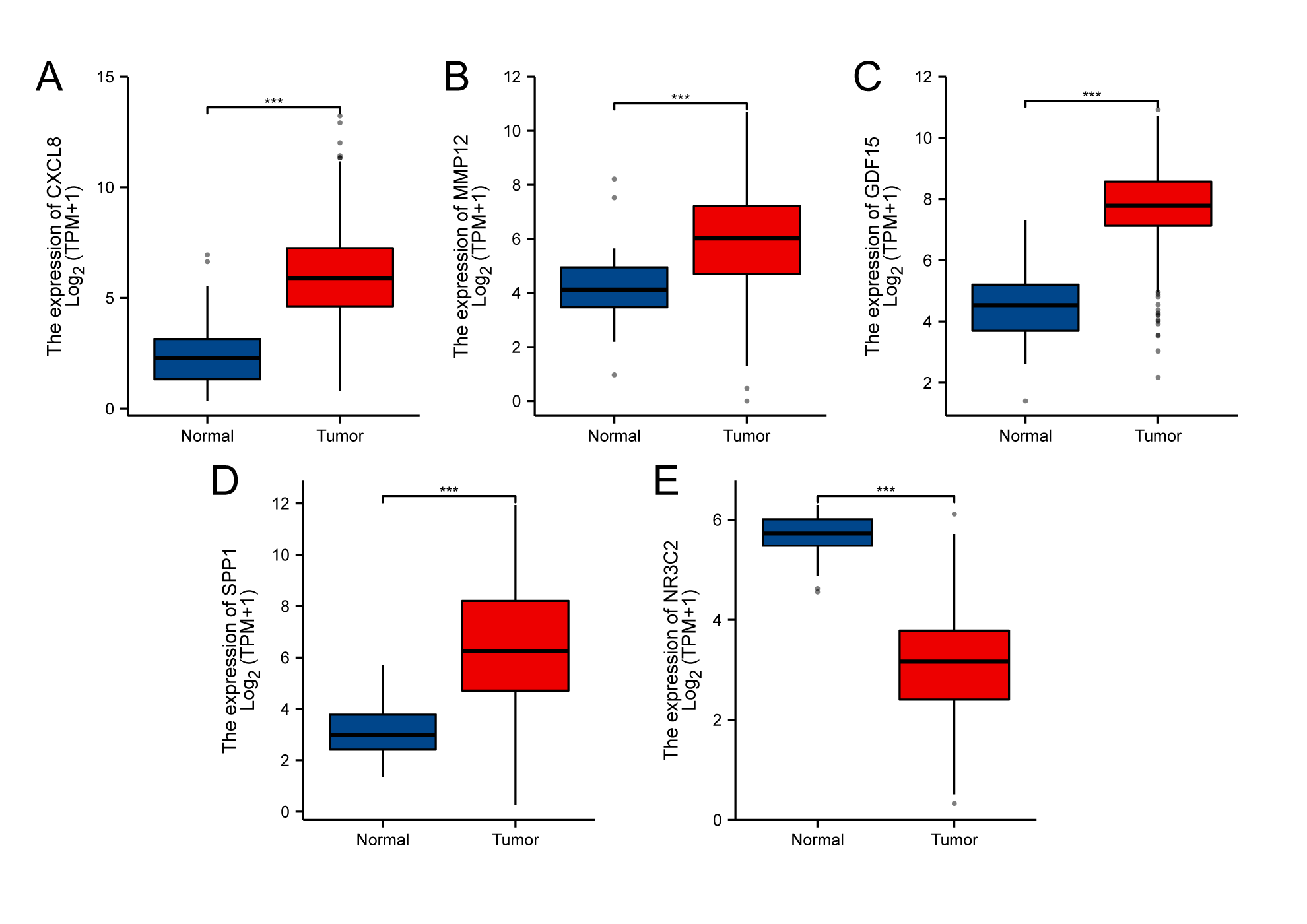

Supplement: Supplementary Figure 1 — The relationship of immune cell infiltration with CXCL8 level in THCA. (A) Overall survival analysis for CXCL8 in CRC patients was performed using the Kaplan–Meier plotter. (B) Survival-dependent receiver operating characteristic (ROC) curve validation of prognostic value of the prognostic index. (C) The correlation of CXCL8 expression level with immune score, stromal score, and ESTIMATE score. (D) Relationships among infiltration levels of 24 immune cell types and CXCL8 expression profiles by ssGSEA. (E) Correlation of CXCL8 expression with immune infiltration level in CRC. *p < 0.05, **p < 0.01, ***p < 0.001. [file Image_1.tif]
