# Supplementary material for: Coagulation- and fibrinolysis-related genes for predicting survival and immunotherapy efficacy in colorectal cancer
Source: Front Immunol. 2022 Nov 30;13:1023908. doi: 10.3389/fimmu.2022.1023908 (PMC9748552; doi:10.3389/fimmu.2022.1023908)
Supplement: Supplementary file 2 [file Table_1.docx]

Supplementary Table1 Detailed GO and KEGG information

| ONTOLOGY | ID | Description | pvalue | p.adjust | qvalue |
| --- | --- | --- | --- | --- | --- |
| BP | GO:0022617 | extracellular matrix disassembly | 6.10E-11 | 3.33E-08 | 2.16E-08 |
| BP | GO:0030198 | extracellular matrix organization | 4.40E-10 | 8.96E-08 | 5.81E-08 |
| BP | GO:0030574 | collagen catabolic process | 4.91E-10 | 8.96E-08 | 5.81E-08 |
| BP | GO:0043062 | extracellular structure organization | 1.30E-09 | 1.77E-07 | 1.15E-07 |
| BP | GO:0032963 | collagen metabolic process | 4.74E-08 | 5.18E-06 | 3.36E-06 |
| BP | GO:0031667 | response to nutrient levels | 6.34E-05 | 0.005779 | 0.003748 |
| BP | GO:0019730 | antimicrobial humoral response | 0.000173 | 0.012638 | 0.008196 |
| BP | GO:0060396 | growth hormone receptor signaling pathway | 0.000213 | 0.012638 | 0.008196 |
| BP | GO:0032461 | positive regulation of protein oligomerization | 0.000231 | 0.012638 | 0.008196 |
| BP | GO:0071378 | cellular response to growth hormone stimulus | 0.000231 | 0.012638 | 0.008196 |
| BP | GO:0006959 | humoral immune response | 0.000254 | 0.012638 | 0.008196 |
| BP | GO:0048545 | response to steroid hormone | 0.000342 | 0.01561 | 0.010124 |
| BP | GO:0060416 | response to growth hormone | 0.000538 | 0.022643 | 0.014684 |
| BP | GO:0007565 | female pregnancy | 0.000655 | 0.025139 | 0.016303 |
| BP | GO:0032459 | regulation of protein oligomerization | 0.000689 | 0.025139 | 0.016303 |
| BP | GO:0097529 | myeloid leukocyte migration | 0.000849 | 0.026656 | 0.017287 |
| BP | GO:0050900 | leukocyte migration | 0.00091 | 0.026656 | 0.017287 |
| BP | GO:0007584 | response to nutrient | 0.000959 | 0.026656 | 0.017287 |
| BP | GO:0007566 | embryo implantation | 0.000969 | 0.026656 | 0.017287 |
| BP | GO:0044706 | multi-multicellular organism process | 0.000997 | 0.026656 | 0.017287 |
| BP | GO:0030595 | leukocyte chemotaxis | 0.001023 | 0.026656 | 0.017287 |
| BP | GO:1904645 | response to amyloid-beta | 0.001086 | 0.027003 | 0.017512 |
| BP | GO:0060135 | maternal process involved in female pregnancy | 0.001522 | 0.036194 | 0.023472 |
| BP | GO:0040014 | regulation of multicellular organism growth | 0.001618 | 0.036866 | 0.023908 |
| BP | GO:0061844 | antimicrobial humoral immune response mediated by antimicrobial peptide | 0.001974 | 0.043199 | 0.028015 |
| CC | GO:0031093 | platelet alpha granule lumen | 0.001497 | 0.030128 | 0.021142 |
| CC | GO:0005788 | endoplasmic reticulum lumen | 0.002202 | 0.030128 | 0.021142 |
| CC | GO:0031091 | platelet alpha granule | 0.002739 | 0.030128 | 0.021142 |
| MF | GO:0004222 | metalloendopeptidase activity | 2.51E-08 | 1.23E-06 | 4.50E-07 |
| MF | GO:0004252 | serine-type endopeptidase activity | 2.29E-07 | 4.74E-06 | 1.73E-06 |
| MF | GO:0008237 | metallopeptidase activity | 4.22E-07 | 4.74E-06 | 1.73E-06 |
| MF | GO:0008236 | serine-type peptidase activity | 4.34E-07 | 4.74E-06 | 1.73E-06 |
| MF | GO:0017171 | serine hydrolase activity | 4.83E-07 | 4.74E-06 | 1.73E-06 |
| MF | GO:0005125 | cytokine activity | 1.11E-06 | 8.70E-06 | 3.18E-06 |
| MF | GO:0004175 | endopeptidase activity | 1.24E-06 | 8.70E-06 | 3.18E-06 |
| MF | GO:0048018 | receptor ligand activity | 2.51E-06 | 1.54E-05 | 5.62E-06 |
| MF | GO:0008009 | chemokine activity | 0.000879 | 0.004787 | 0.001748 |
| MF | GO:0042379 | chemokine receptor binding | 0.001589 | 0.007788 | 0.002844 |
| MF | GO:0005126 | cytokine receptor binding | 0.002001 | 0.008914 | 0.003255 |
| MF | GO:0002020 | protease binding | 0.005829 | 0.023322 | 0.008517 |
| MF | GO:0005178 | integrin binding | 0.006187 | 0.023322 | 0.008517 |
| MF | GO:0008083 | growth factor activity | 0.009296 | 0.029611 | 0.010814 |
| MF | GO:0045236 | CXCR chemokine receptor binding | 0.009903 | 0.029611 | 0.010814 |
| MF | GO:0004866 | endopeptidase inhibitor activity | 0.010652 | 0.029611 | 0.010814 |
| MF | GO:0008239 | dipeptidyl-peptidase activity | 0.010799 | 0.029611 | 0.010814 |
| MF | GO:0030414 | peptidase inhibitor activity | 0.011482 | 0.029611 | 0.010814 |
| MF | GO:0061135 | endopeptidase regulator activity | 0.011482 | 0.029611 | 0.010814 |
| MF | GO:0008191 | metalloendopeptidase inhibitor activity | 0.014374 | 0.035217 | 0.012861 |
| MF | GO:0070064 | proline-rich region binding | 0.016157 | 0.036349 | 0.013275 |
| MF | GO:0061134 | peptidase regulator activity | 0.01632 | 0.036349 | 0.013275 |
| KEGG | hsa05323 | Rheumatoid arthritis | 1.09E-05 | 0.000295 | 0.000215 |
| KEGG | hsa04657 | IL-17 signaling pathway | 1.14E-05 | 0.000295 | 0.000215 |
| KEGG | hsa04060 | Cytokine-cytokine receptor interaction | 0.00096 | 0.016645 | 0.01213 |
| KEGG | hsa05219 | Bladder cancer | 0.001893 | 0.024613 | 0.017936 |
| KEGG | hsa05134 | Legionellosis | 0.003632 | 0.037771 | 0.027526 |
| KEGG | hsa05120 | Epithelial cell signaling in Helicobacter pylori infection | 0.005431 | 0.04707 | 0.034302 |

Supplementary Table 2 Human primers for RT-qPCR

| **Gene** | **Sequence (5'-3')** |
| --- | --- |
| CXCL8 (human) | Forward: ACTGAGAGTGATTGAGAGTGGAC |
|  | Reverse: AACCCTCTGCACCCAGTTTTC |
| MMP12 (Human) | Forward: GATCCAAAGGCCGTAATGTTCC |
|  | Reverse: TGAATGCCACGTATGTCATCAG |
| GDF15 (Human) | Forward: GACCCTCAGAGTTGCACTCC |
|  | Reverse: GCCTGGTTAGCAGGTCCTC |
| SPP1 (human) | Forward: GAAGTTTCGCAGACCTGACAT |
|  | Reverse: GTATGCACCATTCAACTCCTCG |
| NR3C2 (human) | Forward: CGCCGTTGTTAAAAGCCCTAT |
|  | Reverse: GCTGCAAACCGAAGATGTCAT |
| GAPDH (human) | Forward: GGAGCGAGATCCCTCCAAAAT |
|  | Reverse: GGCTGTTGTCATACTTCTCATGG |

Supplementary Table 3 materials

| Material | brand | Province | Country |
| --- | --- | --- | --- |
| TRIzol | Invitrogen |  | USA |
| Synthesis SuperMix | Accurate Biotechnology | Hunan | China |
| Power SYBR® Green | Accurate Biotechnology | Hunan | China |
| RIPA | Beyotime | Shanghai | China |
| PMSF | Beyotime | Shanghai | China |
| 5% nonfat milk | Biotopped | Beijing | China |
| PVDF membranes | Sigma-Aldrich® |  | USA |
| xylene | Tianjin Fuyu Fine Chemical Co | Tianjin | China |
| 3% H2O2 | Solarbio | Beijing | China |
| 3,3'-diaminobenzidine | Solarbio | Beijing | China |

Supplementary Table 4 antibodies

| antibodies | dilution | brand | Province | Country |
| --- | --- | --- | --- | --- |
| CXCL8/IL-8 | 1:500 | Wanleibio | Shenyang | China |
| GAPDH | 1:1000 | Proteintech | Chicago | USA |
| anti-rabbit（WB） | 1:5000 | Proteintech | Chicago | USA |
| anti-mouse（WB） | 1:5000 | Proteintech | Chicago | USA |
| CD3 | ready-to-use | Maixin | Fujian | China |
| CD4 | ready-to-use | Maixin | Fujian | China |
| CD8 | ready-to-use | Maixin | Fujian | China |
| CD20 | ready-to-use | Maixin | Fujian | China |
| CD56 | ready-to-use | Maixin | Fujian | China |
| CD68 | ready-to-use | Maixin | Fujian | China |
| CD163 | ready-to-use | Maixin | Fujian | China |
| anti-rabbit（IHC） | 1:100 | Solarbio | Beijing | China |

Supplementary Table 5 abbreviation

| Abbreviation | Full name |
| --- | --- |
| CRC | Colorectal cancer |
| TCGA | The Cancer Genome Atlas |
| GEO | Gene Expression Omnibus |
| DEGs | differentially expressed genes |
| IRGs | immune-related genes |
| CFRGs | coagulation and fibrinolysis related genes |
| ROC | receiver operating characteristic |
| OS | overall survival |
| IRGPI | Immune-related gene-based prognostic index |
| ICIs | immune checkpoint inhibitors |
| CIMP | CpG-island methylator phenotype |
| MSI | microsatellite instability |
| CIN | chromosomal instability |
| CMS | consensus molecular subtypes |
| CTLA-4 | cytotoxic T-lymphocyte-associated protein 4 |
| PD-1 | programmed cell death protein 1 |
| PD-L1 | programmed cell death 1 ligand 1 |
| KEGG | Kyoto Encyclopedia of Genes and Genomes |
| LASSO | Least Absolute Shrinkage and Selection Operator |
| GO | Gene Ontology |
| BP | biological process |
| CC | cellular component |
| MF | molecular function |
| RIPA | Radio immunoprecipitation assay |
| AUC | area under curve |
| TILs | tumor‐infiltrating lymphocytes |
| VTE | venous thromboembolism |
| NK | natural killer |
| CEA | carcinoembryonic antigen |
| CXCL8 | C-X-C motif chemokine ligand 8 |
| MMP12 | matrix metallopeptidase 12 |
| GDF15 | growth differentiation factor 15 |
| SPP1 | secreted phosphoprotein 1 |
| NR3C2 | nuclear receptor subfamily 3 group C member 2 |
